# Impact of mechanical power and positive end expiratory pressure on central vs. mixed oxygen and carbon dioxide related variables in a population of female piglets

**DOI:** 10.14814/phy2.15954

**Published:** 2024-02-16

**Authors:** Antonio Fioccola, Tommaso Pozzi, Isabella Fratti, Rosmery Valentina Nicolardi, Federica Romitti, Mattia Busana, Francesca Collino, Luigi Camporota, Konrad Meissner, Onnen Moerer, Luciano Gattinoni

**Affiliations:** ^1^ Department of Anesthesiology University Medical Center Göttingen Göttingen Germany; ^2^ Department of Health Sciences, Section of Anaesthesiology, Intensive Care and Pain Medicine University of Florence Florence Italy; ^3^ Department of Health Sciences University of Milan Milan Italy; ^4^ IRCCS San Raffaele Scientific Institute Milan Italy; ^5^ Department of Surgical Sciences University of Turin Turin Italy; ^6^ Department of Adult Critical Care Guy's & St Thomas' NHS foundation Trust London UK; ^7^ Centre for Human & Applied Physiological Sciences, School of Basic & Medical Biosciences King's College London London UK

**Keywords:** ARDS, central versus mixed gap, oxygen saturation, carbon dioxide, intrathoracic pressures, mechanical power, oxygen saturation, ScvO_2_, septic shock, SvO_2_

## Abstract

**Introduction:**

The use of the pulmonary artery catheter has decreased overtime; central venous blood gases are generally used in place of mixed venous samples. We want to evaluate the accuracy of oxygen and carbon dioxide related parameters from a central versus a mixed venous sample, and whether this difference is influenced by mechanical ventilation.

**Materials and Methods:**

We analyzed 78 healthy female piglets ventilated with different mechanical power.

**Results:**

There was a significant difference in oxygen‐derived parameters between samples taken from the central venous and mixed venous blood (S$\bar{v}$O_2_ = 74.6%, ScvO2 = 83%, *p* < 0.0001). Conversely, CO2‐related parameters were similar, with strong correlation. Ventilation with higher mechanical power and PEEP increased the difference between oxygen saturations, (Δ[ScvO2−S$\bar{v}$O_2_ ] = 7.22% vs. 10.0% respectively in the low and high MP groups, *p* = 0.020); carbon dioxide‐related parameters remained unchanged (*p* = 0.344).

**Conclusions:**

The venous oxygen saturation (central or mixed) may be influenced by the effects of mechanical ventilation. Therefore, central venous data should be interpreted with more caution when using higher mechanical power. On the contrary, carbon dioxide‐derived parameters are more stable and similar between the two sampling sites, independently of mechanical power or positive end expiratory pressures.

## 1 INTRODUCTION

Oxygen (O_2_) and carbon dioxide (CO_2_) related variables are important physiological parameters used to monitor critically ill patients. Blood samples taken from the mixed venous blood—using a pulmonary artery catheter (PAC), are considered more representative of the O_2_ consumption and CO_2_ production of the upper body (including brain and myocardium) and lower body, and therefore a more accurate estimation of the key physiological variables utilized in the management of critically ill patients: cardiac output, pulmonary vascular resistances, pulmonary arterial pressures, mixed venous oxygen saturation (S$\bar{v}$O_2_) and mixed venous carbon dioxide partial pressure (P$\bar{v}$CO_2_).

Amongst the oxygen‐derived variables, S$\bar{v}$O_2_ and oxygen extraction ratio (O_2_ER), that is, the ratio of oxygen consumption (VO_2_) to oxygen delivery (DO_2_) (Vincent, 1996) can guide the hemodynamic management in in low oxygen delivery states (i.e., low perfusion and/or distributive shock) (Cain, 1977; Schumacker & Cain, 1987). The main feature of O_2_ER critical point (O_2_ER_crit_) is its ability to predict DO_2_ inadequacy earlier and more accurately than an increase in arterial lactate, which might be affected by a variety of other factors independently from oxygen transport (Kraut & Madias, 2014).

Additional variables such as the Arteriovenous difference of oxygen content (ΔContO_2_), the veno‐arterial PCO2 difference (ΔPCO_2_) and their ratio (ΔPCO_2_/ΔContO_2_) have been largely studied as indices of perfusion adequacy in critically ill patients (Cuschieri et al., 2005; Mallat et al., 2016; Jakob et al., 2015; Mekontso‐Dessap et al., 2002; Monnet et al., 2013). A clinical cut‐off value of 6 mmHg of ΔPCO_2_ has been proposed to identify low flow states (Mallat, Lemyze, Tronchon, et al., 2016), while the ΔPCO_2_/ΔContO_2_ has been found to be a better perfusion as indicator compared to ΔPCO_2_ alone as it will take into account changes in O_2_ consumption (VO_2_) (Mallat, Lemyze, Meddour, et al., 2016; Nassar et al., 2021).

Despite the potential usefulness of these physiological parameters, the use of a PAC has progressively declined (Connors et al., 1996; Gore et al., 1987; Ikuta et al., 2017) and substituted by less invasive alternatives (Arias‐Ortiz & Vincent, 2023; Vincent et al., 2008) involving sampling from a central venous catheter (CVC).

Although several studies have suggested that oximetry obtained from the central venous blood can be usefully employed in place of mixed venous (Dueck et al., 2005; Maddirala & Khan, 2010; Pope et al., 2010; Reinhart et al., 2004), the absolute values may vary substantially in clinical settings. In addition, data on the difference between the central and the mixed venous sample for PCO_2_ and derived variables (ΔPCO_2_) is lacking.

Given the potential diagnostic and therapeutic implications of using data from central venous catheters, it is important to understand whether the differences in oxygen‐related parameters (e.g., saturation) applies also to CO_2_ tensions, and whether mechanical ventilation setting can affect the changes and in which circumstances.

Therefore, the aim of this study was to investigate, in a large sample of 78 mechanically ventilated piglets, the differences in oxygen and carbon dioxide variables obtained from central and mixed venous samples. Moreover, we aimed to assess whether these differences are influenced by the mechanical ventilation settings and intrathoracic pressures.

## 2 MATERIALS AND METHODS

### 2.1 Study design

We performed a retrospective analysis on 78 mechanically ventilated female piglets under general anesthesia in the context of two previous ventilator‐induced lung injury (VILI) studies (Collino et al., 2019; Vassalli et al., 2020). Protocols were approved by the local authorities (Niedersächsisches Landesamt für Verbraucherschutz und Lebensdmittelsicherheit: LAVES; Oldenburg, Niedersachsen, Germany. Study 1 (Collino et al., 2019): date of approval: 09/01/2017; assigned number 16/2223. Study 2 (Vassalli et al., 2020): date of approval: 24/05/2018; assigned number: 18/2795) and were performed in accordance with the European Guidelines 2010/63 and the 1975 Helsinki declaration. Each experiment lasted 48 h and blood samples were collected every 6 h. After induction of general anesthesia an orotracheal intubation was performed. A CVC and a PAC were inserted via the external jugular vein, while an arterial catheter was placed through the femoral artery, under ultrasound guidance. The PAC tip location was confirmed by observing a typical pulmonary artery wave and wedge pressure during its placement, using 1 mL of air to fill the distal balloon. The CVC tip location at the junction between the superior vena cava and the right atrium was confirmed during the autopsy, which was routinely practiced at the end of each experiment. Pigs were sedated with propofol, sufentanil, and midazolam and initially ventilated with standardized settings with tidal volumes of 7 mL/kg, a respiratory rate to obtain an end‐tidal carbon dioxide partial pressure (EtCO_2_) between 40 and 50 mmHg, a positive end‐expiratory pressure (PEEP) of 4 cmH_2_O and an inspired oxygen fraction of 0.40. After a 30 min stabilization period, a wide range of mechanical power (MP) (from 3.9 to 60.9 J/min) was applied, using different combinations of tidal volume, respiratory rate and PEEP and these were kept for the duration of the experiment.

### 2.2 Measurements

Blood samples were collected slowly and simultaneously from all the vascular catheters (arterial, CVC and PAC, noted as subscripts as a, cv and $\bar{v}$, respectively), and analyzed for oxygen and carbon dioxide‐related analytes: oxygen (PO_2_) and carbon dioxide (PCO_2_) partial pressures, oxygen saturation (SO_2_), hemoglobin concentration (Hb) and pH (GEM Premier 5000, Werfen). Oxygen‐ and carbon‐dioxide‐derived variables (VO_2_, O_2_ER, venous admixture, bicarbonate concentration—[HCO_3_^−^]–and arterial–venous carbon dioxide gradient−ΔPCO_2_) using mixed or central venous oxygen saturation were calculated using standard formulas. The complete set of equations used is available in the Data S1.

### 2.3 Stratification according to mechanical power

To evaluate the possible influence of the intensity of mechanical ventilation on the relationship between central and mixed venous oxygen‐and carbon dioxide‐related analyses and derived variables, piglets were divided according to the applied median of respiratory system mechanical power (MP) (Gattinoni et al., 2016) during the experimental phase into low (≤23.9 J/min) and high MP (>23.9 J/min) groups. The equation used to calculate the MP is available in the Data S1 (10.6084/m9.figshare.24271156).

### 2.4 Stratification according to mechanical power components

To investigate the relative contribution of each mechanical power component (i.e., tidal volume, respiratory rate and PEEP) during the experimental phase, the whole study population was subsequently divided according to (1) the median value of applied tidal volume into low (≤14.3 mL/kg) and high (>14.3 mL/kg) tidal volume; (2) the median value of applied respiratory rate into low (≤30 bpm) and high (>30 bpm) respiratory rate and (3) the median value of applied PEEP into low (≤5 cmH_2_O) and high (>5 cmH_2_O) PEEP.

### 2.5 Statistical analysis

Continuous variables are expressed as mean ± standard deviation or median [interquartile range], as appropriate. In the whole population, the relationship between oxygen‐and carbon dioxide‐related analytes and derived variables obtained by central and mixed venous samples was assessed by a linear mixed effect model, with the mechanical power as a between fixed effect, time as a within fixed effect and piglets as random effect, by computing the concordance correlation coefficient (CCC) (Zhang, 2022) and by a repeated‐measures Bland–Altman analysis. Differences between low and high MP groups in variables at baseline and during the experimental phase (taken as the average values for each pig of all experimental timepoints) were assessed by Student's *t* or Wilcoxon‐Mann–Whitney *U* test, as appropriate. A *p* <0.050 was considered as significant. All statistical analyses were performed with R 4.2.3 (R Foundation for Statistical Computing, Vienna, Austria).

## 3 RESULTS

### 3.1 Oxygen‐ and CO_2_‐related analytes and derived variables in the whole population

**FIGURE 1:**
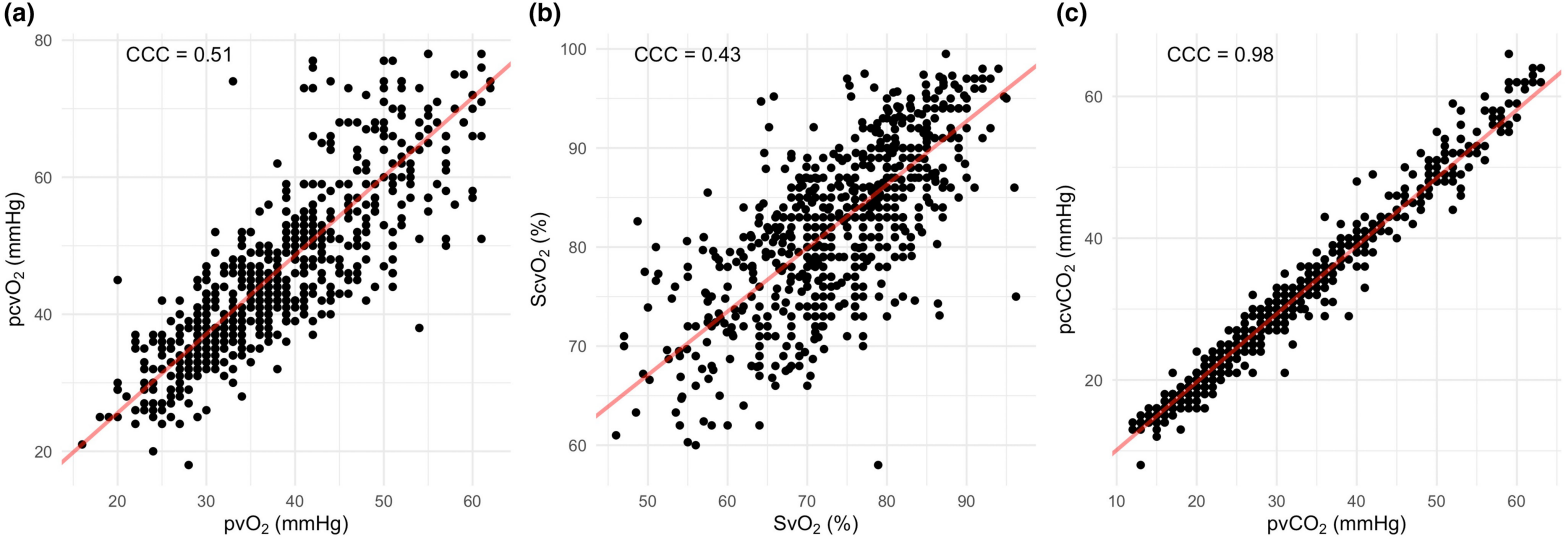
Linear regressions between oxygen and carbon‐dioxide related variables in a mixed and in a central venous blood sample. P$\bar{v}$O_2_ and PcvO_2_ showed a moderate correlation (CCC = 0.51), as well as S$\bar{v}$O_2_ and ScvO_2_ (CCC = 0.43). P$\bar{v}$CO_2_ and PcvCO_2_ showed a very strong correlation (CCC 0.98). CCC, concordance correlation coefficient; mmHg, millimeters of mercury; PvO_2_, mixed venous blood oxygen partial tension; P$\bar{v}$CO_2_, mixed venous blood carbon dioxide partial tension; PcvO_2_, central venous blood oxygen partial tension; PcvCO_2_, central venous blood carbon dioxide partial tension; S$\bar{v}$O_2_, mixed venous blood oxygen saturation; ScvO_2_, central venous blood oxygen saturation.

**FIGURE 2:**
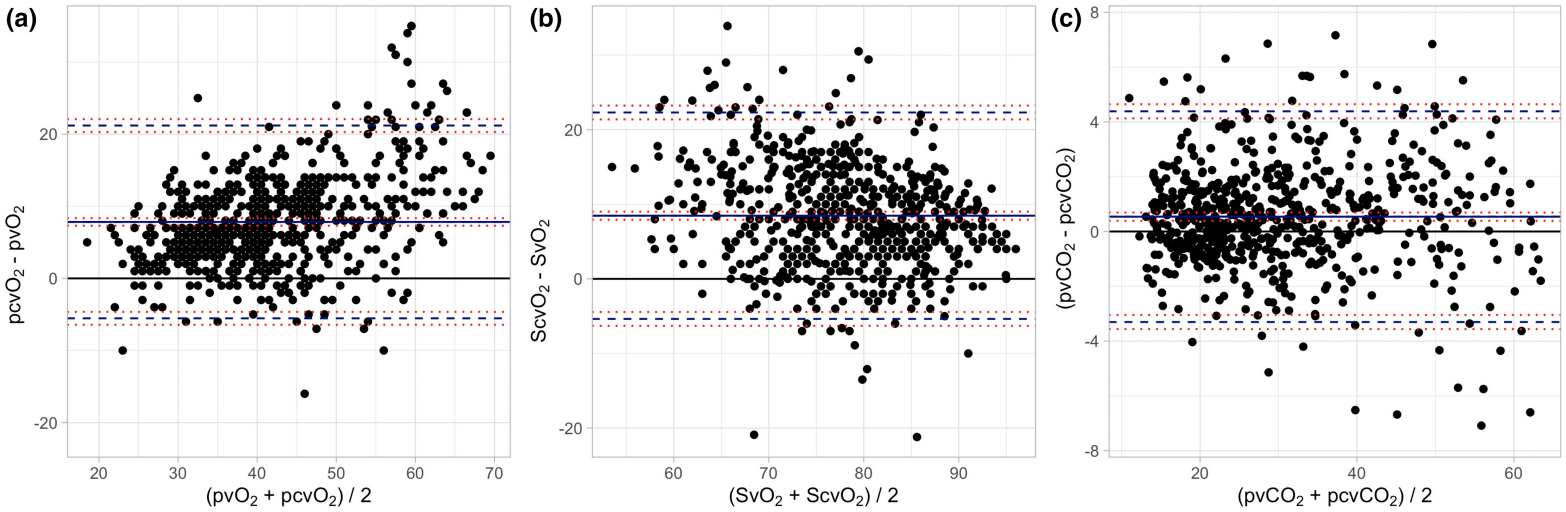
Bland–Altman analysis between oxygen and carbon‐dioxide related variables in a mixed and in a central venous blood sample. Panel a: mean bias = 7.82 [7.30–8.35]; upper LOA = 21.19 [20.29–22.09]; lower LOA = −5.53 [−6.43 to‐4.64]. Panel b: mean bias = 8.47 [7.92–9.01]; upper LOA = 22.30 [21.37–23.23]; lower LOA = −5.36 [−6.29 to‐4.44]. Panel c: mean bias = 0.54 [0.39–0.69]; upper LOA = 4.38 [4.12–4.64]; lower LOA = −3.30 [−3.56 to‐3.04]. LOA, Level of Agreement; P$\bar{v}$O_2_, mixed venous blood oxygen partial tension; P$\bar{v}$CO_2_,mixed venous blood carbon dioxide partial tension; PcvCO_2_, central venous blood carbon dioxide partial tension; PcvO_2_, central venous blood oxygen partial tension; S$\bar{v}$O_2 =_ mixed venous blood oxygen saturation; ScvO_2_, central venous blood oxygen saturation. 95% confidence intervals are shown in square brackets.

In Figure 1 we report linear regressions of O_2_ and CO_2_‐related analytes in the whole study population. Bland and Altman analyses are shown in Figure S1. As shown, PcvO_2_ and P$\bar{v}$O_2_ were moderately correlated (CCC = 0.51; Figure 1, panel a), with a mean bias of 7.8 mmHg (upper Level of Agreement, LOA = 21.19, lower LOA = −5.53; Figure 2, panel a). A similar behavior was observed for oxygen saturation (Figure 1, panel b and Figure 2, panel b). Both PO_2_ and SO_2_ were statistically different between central and mixed venous samples (*p* < 0.0001; Table 1). Conversely, PcvCO_2_ and P$\bar{v}$CO_2_ showed a stronger correlation (CCC = 0.98; Figure 1, panel c) with a mean bias of 0.5 mmHg (upper LOA = 4.38, lower LOA = −3.30; Figure 2, panel c), resulting non statistically different (*p* = 0.26; Table 1).

**TABLE 1:** Mixed linear models of oxygen and carbon dioxide related analytes in the whole population, considering the baseline and the experimental phase, from a mixed and a central venous sample.

Variable	Mixed venous sample	Central venous sample	*p*
pO_2_ (mmHg)	38 [32–45]	45 [38–54]	**< 0.0001**
SO_2_ (%)	74.6 [67–81]	83 [76.6–89]	**< 0.0001**
pCO_2_ (mmHg)	28 [21–40]	28 [21–39]	0.264
[HCO_3_^−^] (mEq/L)	25.3 [20.5–30.4]	24.7 [20.1–29.8]	0.191
VO_2_ (mL/min)	95.3 [76.1–118.0]	69.2 [54.2–84.9]	**< 0.0001**
O_2_ER (%)	28.6 [22.5–35.8]	17.1 [11–23]	**< 0.0001**
**Δ**ContO_2_ (mL/dL)	3.1 [2.3–3.8]	2.2 [1.5–2.9]	**< 0.0001**
Qva/Qt (%)	3.55 [2.55–5.10]	4.7 [3.8–7.4]	**< 0.0001**
**Δ**PCO_2_ (mmHg)	7 [5–8]	6 [4–8]	0.155

*Note*: Values are shown as median and interquartile range, reported in square brackets. To assess differences amongst the mixed and the central venous sample, a linear mixed model has been performed, with the pigs as random effect. All the shown *p*‐values are both‐sided. All the bold values provided in table 1 are < 2 * 10^‐16^.

Abbreviations: ΔContO_2_, arterio‐venous difference of oxygen content; HCO_3_^−^, bicarbonates concentration; mmHg, millimitres of mercury; mEq/L, milliequivalents per liters; mL/min, milliliters per min; %, percentage; mL/dL, milliliters per deciliter; O_2_ER, oxygen extraction ratio; ΔPCO_2_, veno‐arterial carbon dioxide partial tension difference; pCO_2_, venous blood carbon dioxide partial tension; PO_2_, venous blood oxygen partial tension; SO_2_, mixed venous blood oxygen saturation; VO_2_, oxygen consumption; Qva/Qt, pulmonary venous admixture.

In Figure S1 we show the regression and the Bland–Altman analysis for [HCO_3_^−^], that resulted to be highly correlated (CCC = 0.98) and not statistically different in the central and mixed venous sample, with a similar behavior to PCO_2_ (*p* = 0.19, Table 1).

In Figure S2 we report linear regressions of oxygen‐derived variables in the whole study population. While VO_2_ and O_2_ER from central and mixed venous samples showed a poor correlation (CCC = 0.35 and CCC = 0.27, Figure S2, panel a and b respectively), ΔContO_2_ and Qva/Qt showed better correlations (CCC = 0.42 and CCC = 0.76, respectively). However, as shown in Table 1, all oxygen‐derived variables were statistically different between central and mixed venous samples. At the opposite, ΔPCO_2_ was similar when computed with a central or a mixed venous sample (*p* = 0.15, Table 1), showing a high correlation (CCC = 0.71 Figure S3, panel a).

### 3.2 Mechanical power groups

#### 3.2.1 Baseline values

The value used to separate low versus high MP (i.e., the median MP in our study population during the experimental phase) was 23.9 J/min, resulting in two groups (low and high mechanical power) with 39 piglets each, with a similar mean weight of 23.8 ± 2.2 and 23.9 ± 2.1 kg, respectively. In Table S1 we report the baseline values of the main zoometric, respiratory and hemodynamic variables, including all the determinants of DO_2_, VO_2_, ScvO_2_, and S$\bar{v}$O_2_, according to mechanical power groups. As shown, no statistical difference was found between the groups at baseline.

#### 3.2.2 Experimental phase values

##### Differences between the two groups of mechanical power

In Table 2 we report the main O_2_ and CO_2_ related analytes and derived variables calculated from the mixed and the central venous sample in the two groups of Low and High Mechanical Power during the experimental phase. All relevant hemodynamic characteristics according to mechanical power groups are also reported.

**TABLE 2:** Piglets' hemodynamic variables during the whole experimental phase. For each piglet, the mean value of all the measurement collected during the experiment (from timepoint 30 min to 48 h) was considered.

Variable (experimental phase)	Low mechanical power (< 23.9 J/min)	High mechanical power (>23.9 J/min)	*p*
Cardiac output (L/min)	3.1 [2.8–3.5]	3.16 [2.6–3.58]	0.847
Hb (g/dL)	7.54 ± 0.93	7.16 ± 0.99	0.087
SaO_2_ (%)	100 [98.8–100]	100 [98.9–100]	0.982
S$\bar{v}$O_2_ (%)	75.3 [73.2–78.9]	70.8 [65.7–74.6]	**<0.001**
ScvO_2_ (%)	82.4 [78.2–85.6]	81.6 [78.4–85.3]	0.558
Δ (ScvO_2_−S$\bar{v}$O_2_)	7.22 [3.9–10.9]	10.0 [5.5–14.2]	**0.020**
PaO_2_ (mmHg)	218 [201–226]	230 [224–238]	**<0.001**
P$\bar{v}$O_2_ (mmHg)	39.6 [34.9–43.7]	32.8 [29.6–42.2]	**0.005**
PcvO_2_ (mmHg)	46.9 [41.5–50.7]	45.3 [39–52.8]	0.354
Δ(PcvO_2_−P$\bar{v}$O_2_) (mmHg)	6.9 ± 3.9	9.4 ± 5.0	**0.023**
PaCO2 (mmHg)	24.3 [20.7–33.7]	18 [14.2–25]	**<0.001**
P$\bar{v}$CO2 (mmHg)	31.4 [26.6–41.1]	24 [18.8–30.4]	**0.003**
PcvCO2 (mmHg)	30.7 [26.4–38.8]	23.8 [18.3–31.4]	**0.003**
Δ(P$\bar{v}$CO_2_−PcvCO_2_) (mmHg)	0.5 [−0.3–0.9]	0.5 [0.1–0.9]	0.344
[HCO_3_^−^_mixed_] (mEq/L)	26.4 [23.5–28.3]	24.3 [20–26.8]	**0.008**
[HCO_3_^−^_central_] (mEq/L)	25.9 [23.0–27.8]	23.6 [19.9–26.0]	**0.003**
Δ([HCO_3_^−^_mixed_]‐ [HCO_3_^−^_central_]) (mEq/L)	0.41 [−0.04–0.66]	0.55 [0.13–1.23]	0.113
DO_2_ (mL/min)	345 [295–384]	347 [260–385]	0.303
VO_2_ (mL/min)	90.5 [73.9–117]	94.7 [84–118]	0.329
O_2_ER_mixed_ (%)	28 ± 5.3	32.9 ± 6.2	**<0.001**
O_2_ER_central_ (%)	17.5 ± 4.5	18.7 ± 5.9	0.696
Δ (O_2_ER_mixed_−O_2_ER_central_) (%)	10.6 ± 4.2	14.2 ± 5.2	**0.002**
Qva/Qt_mixed_ (%)	3.7 [2.6–5]	3.1 [2.5–5.2]	0.636
Qva/Qt_central_ (%)	4.7 [4.1–7.4]	4.7 [3.7–7.7]	0.874
Δ (Qva/Qt_mixed_−Qva/Qt_central_) (%)	1.2 [0.6–2]	1.5 [0.8–2.6]	0.336
MAP (mmHg)	71.4 [67.4–74.9]	72.2 [68.1–80.8]	0.254
CVP (mmHg)	8.94 [6.66–12]	11.5 [8.33–15.7]	**0.03**
Mean pulmonary pressure (mmHg)	20.9 [17.8–27.2]	26.4 [23.7–33.8]	**0.002**
Cumulative fluid balance (L)	2.76 [0.63–3.93]	2.45 [0.13–5.10]	0.401
Left cardiac work (J/min)	25 [21.8–27.8]	24.3 [21.7–29.4]	0.856
Right cardiac work (J/min)	5.12 [4.00–6.75]	7.43 [5.34–9.02]	**0.009**
Overall cardiac work (J/min)	30.3 [26.6–35]	32.6 [27.6–37.4]	0.187

*Note*: Means +/− SDs were reported for variables with a normal distribution, while medians and interquartile ranges (in square brackets) were reported for non‐normally distributed variables.

Abbreviations: cmH_2_O, centimeters of water; CVP, central venous pressure; dL, deciliters; g, grams; J/min: Joules per minute; L, liters; MAP, mean arterial pressure; min, minutes; mmHg: millimeters of mercury; O_2_ER_mixed_, oxygen extraction ratio calculated with the mixed venous sample; O_2_ER_central_, oxygen extraction ratio measured with the central venous sample; Δ (O_2_ER_mixed_ – O_2_ER_central_), difference between oxygen extraction ratio calculated with a mixed and a central venous sample; Δ (S_c_vO_2_ – S$\bar{v}$O_2_), difference between the central and the mixed venous oxygen saturation; S$\bar{v}$O_2_, mixed venous oxygen saturation; S_c_vO_2_, central venous oxygen saturation.

As shown, the difference between central and mixed venous oxygen saturation, Δ(ScvO_2_−S$\bar{v}$O_2_), was significantly higher in piglets ventilated with higher MP compared to piglets ventilated with lower MP (10.0 [5.5–14.2] vs. 7.2 [3.9–10.9] %, *p* = 0.020). Similarly, Δ(PcvO_2_−P$\bar{v}$O_2_) was higher in piglets ventilated with higher MP (6.9 ± 3.9 vs. 9.4 ± 5.0, *p* = 0.023).

Conversely, the difference between the central and the mixed venous sample of carbon dioxide partial tension, Δ(P$\bar{v}$CO_2_−PcvCO_2_), was not affected by the mechanical power (0.5 [−0.3–0.9] vs. 0.5 [0.1–0.9], *p* = 0.344).

In piglets ventilated with higher MP, PaO_2_ was higher (*p* < 0.001) while P$\bar{v}$O_2_ and S$\bar{v}$O_2_ were significantly lower (32.8 [29.6–42.2] vs. 39.6 [34.9–43.7] mmHg, *p* = 0.005 and 70.8 [65.7–74.6] vs. 75.3 [73.2–78.9] %, *p* < 0.001, respectively). The ScvO_2_ was not statistically different between the two groups (*p* = 0.558). Considered individually, DO_2_ and VO_2_ did not differ amongst the two groups. The O_2_ER_mixed_ was significantly higher in the high MP group, while the O_2_ER_central_ did not change. No difference was found between the overall cardiac work in the two groups (*p* = 0.187). Consequently, the difference between O_2_ER_mixed_ and O_2_ER_central_, Δ (O_2_ER_mixed_−O_2_ER_central_), resulted to be higher in piglets ventilated with higher mechanical power (*p* = 0.002).

In Table S2, we show the same results from a mixed linear model, in which we considered the same variables as continuous and dependent from the mechanical power, the time and their interaction.

##### Experimental time‐course

**FIGURE 3:**
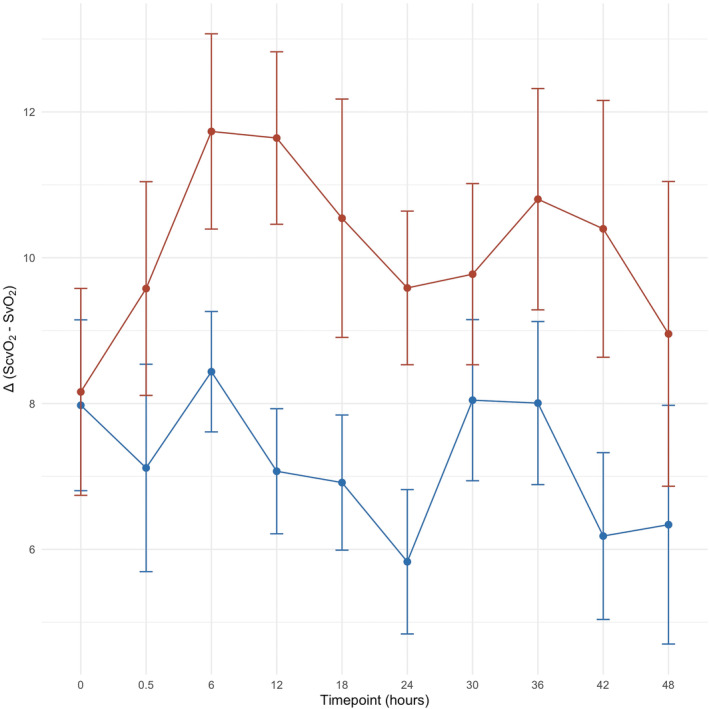
Time course of the Δ(S_c_vO_2_ – S$\bar{v}$O_2_) in the two groups of mechanical power during the whole experimental phase. Mean +/− SD of piglets in the lower MP group are represented in blue, while means +/− SD of piglets in the higher MP group are shown in red. Excluding the baseline timepoint, from the beginning of the experimental phase through the end of the experiment, the Δ(S_c_vO_2_−S$\bar{v}$O_2_) in the two groups of mechanical power was constant, without any effect of the time (*β* = 0.006, CI = [−0.07–0.06], *p* = 0.849). It was constantly different after setting the mechanical power (from timepoint 0.5 h towards 48 h). X‐axis: experimental timepoints, expressed as hours from the beginning of the experiment. Y‐axis: Δ(S_c_vO_2_−S$\bar{v}$O_2_), difference between central and mixed venous sample oxygen saturation.

In Figure 3 we report the time course of the Δ (S_c_vO_2_−S$\bar{v}$O_2_). As shown, the difference between the two groups was significant but constant throughout the whole experiment. Once excluded the baseline, the linear mixed model showed no effect of time in influencing the difference between central and mixed venous oxygen saturation in the two groups of power (*β* = 0.006, CI = [−0.07–0.06], *p* = 0.849).

**FIGURE 4:**
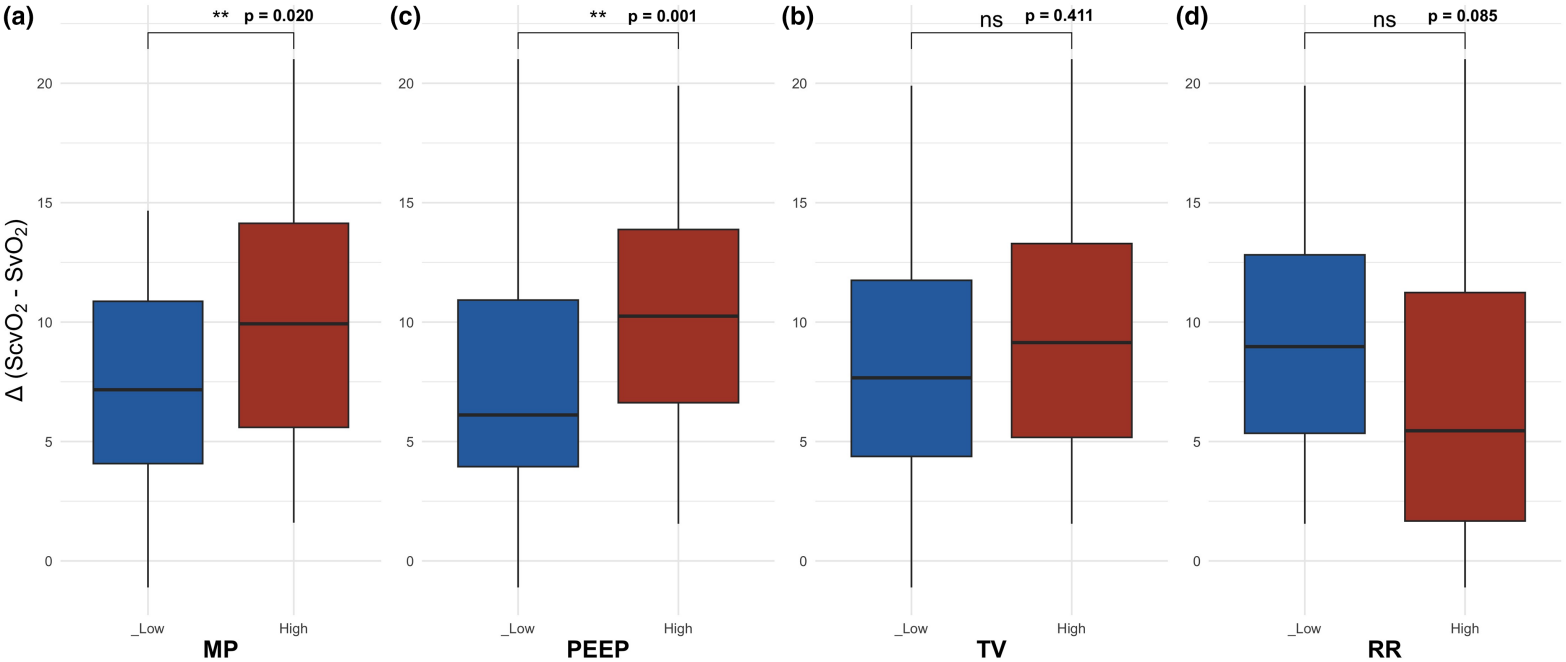
Δ(S_c_vO_2_−S$\bar{v}$O_2_) in the two groups of MP, PEEP, TV and RR. Blue and red boxplots refer respectively to piglets ventilated with low and high mechanical power. MP, mechanical power; PEEP, positive end‐expiratory pressure; TV, tidal volume; RR, respiratory rate; Δ(S_c_vO_2_−S$\bar{v}$O_2_), difference between central and mixed venous blood oxygen saturation. For numerical details, see also Table S3 in the supplementary materials.

Δ (S_c_vO_2_−S$\bar{v}$O_2_) and mechanical power determinants. In Figure 4 and in Table S2 we report the Δ (S_c_vO_2_−S$\bar{v}$O_2_) as a function of the mechanical power and its components. As shown, the only factor associated with Δ(S_c_vO_2_−S$\bar{v}$O_2_) was PEEP, while no associations were found with tidal volume and respiratory rate.

##### Mechanical power effect on O_2_ and CO_2_ related analytes

In Figure S5 and in Table S4 we show the effect of mechanical power of O_2_ and CO_2_ related parameters. As reported, the difference between every O_2_‐related parameter sampled in the central versus the mixed venous sample increases in pigs ventilated with high mechanical power. Conversely, there is no increase in difference between CO_2_ related variables from a central and a mixed venous sample. Numerical values are reported in Table 2.

## 4 DISCUSSION

The main finding of this study was that oxygen saturation, PO_2_ and oxygen content were consistently higher in central venous blood than in the mixed venous blood, while PCO_2_ and bicarbonate concentration were similar in the two sampling sites. In addition, the differences between central venous and pulmonary artery in terms of oxygen‐related variables increased in animals ventilated at higher mechanical power (particularly with PEEP), while PCO_2_ and CO_2_‐related variables remained similar regardless of the applied MP and PEEP. Consequently, oxygen‐derived variables (e.g., VO_2_, O_2_ER and venous admixture) were significantly affected by sampling location and by ventilatory settings (i.e., PEEP), while CO_2_‐derived variables were not.

Since Connors et al. (Connors et al., 1996) reported that the use of Swan‐Ganz catheter was not associated with clear outcome benefit, the routine use of PAC progressively declined and, nowadays, it is only rarely used in clinical practice. Consequently, central venous blood gases have been progressively used in critical care as a surrogate, particularly to monitor possible variations in hemodynamics (cardiac output) or metabolism (VO_2_) and their response to interventions. However, the possible discrepancy between central venous and pulmonary artery samples in terms of oxygen derived variables is increasingly recognized.

The role of venous SO_2_ (either S_c_vO_2_ or S$\bar{v}$O_2_) and its prognostic value has been widely reported in the literature. Shepherd et al. (Shepherd & Pearse, 2009) highlights its importance in the perioperative setting, where low values of S_c_vO_2_ and S$\bar{v}$O_2_ are related to poor post‐operative prognosis. Pearse et al. showed that a S_c_vO_2_ lower than 64.4% was associated with worse prognosis in major non‐cardiac surgery (Pearse et al., 2005). Krauss et al. found a similar cutoff for S$\bar{v}$O_2_in cardiac surgery (Krauss et al., 1975). However, a consensus on a single value has not been reached yet, neither on the interchangeability of the central and the mixed venous sample. This mainly depends from the heterogeneity of the patients included in the studies, the context and the consequent variable relationship of the difference between S_c_vO_2_ and S$\bar{v}$O_2_ reported in the literature. While some studies, similarly to our findings, show a higher value of S_c_vO_2_ when compared to S$\bar{v}$O_2_, especially in patients with hemodynamic instability, shock or under general anesthesia (El Masry et al., 2009; Maddirala & Khan, 2010; Reinhart et al., 2004), others do not show any difference (Martin et al., 1992) or sometimes find a central venous oxygen saturation lower than the mixed sample (Dahn et al., 1988). The main mechanism proposed to explain this phenomenon is the blood redistribution to the upper body in conditions of hemodynamic instability, that would increase the O_2_ER of the lower body when compared to the central nervous system (Shepherd & Pearse, 2009).

Several factors may have contributed to the higher SO_2_ in central venous than in pulmonary arterial blood in our study. The most relevant is likely the anatomical location; indeed, the central venous catheter collects blood mainly from the superior vena cava territory, whereas the contribution of blood returning from the splanchnic territory to the inferior vena cava and from the heart through the coronary sinus is minor. Obviously, blood is completely mixed in the pulmonary artery and the different organs contribute to S$\bar{v}$O_2_ depending on their oxygen consumption. Of note, in normal conditions, oxygen extraction in the myocardium is high and therefore the effluent venous blood has a very low saturation (Bloos & Reinhart, 2005). Similarly, the inferior vena cava normally carries blood with a lower oxygen saturation than the superior vena cava in anesthetized patients, where the brain's oxygen consumption is greatly reduced, as in critical illness (Chawla et al., 2004; El Masry et al., 2009; Lee et al., 1972; Reinhart et al., 2004; Slupe & Kirsch, 2018) Therefore, organ differences in oxygen consumption and the incomplete mixing between blood returning from superior vena cava, inferior and coronary sinus could account for the observed differences. In our study, the increase in difference observed with higher PEEP might be explained by the increased impedance to the venous return from the splanchnic area, with venous congestion and greater oxygen desaturation in blood returning from kidney, bowel and liver. Interestingly, tidal volume did not have the same effect of PEEP on increase of difference of oxygen related variables between the central and the mixed venous sample (Table S3). A possible explanation might be that cyclic increase/decrease of airway pressures have not the same impact of continuous positive airway pressures on intrathoracic circulation, thus not influencing the Δ (ScvO_2_−S$\bar{v}$O_2_) (%).

A possible model to test the differences between the central and mixed venous oxygen saturation accounting for different oxygen consumptions and different contributions of the inferior and superior vena cava as well as of coronary sinus is reported in the Data S1.

Oxygen differences between central venous and pulmonary artery samples have been largely studied (El Masry et al., 2009; Reinhart et al., 1986; Reinhart et al., 2004). Differences of CO_2_‐related variables between mixed and central venous blood have been less investigated and showed conflicting results in different settings in terms of interchangeability between mixed and central venous sample (Ho et al., 2007; Tsaousi et al., 2010). To our knowledge, this is the first analysis considering at the same time oxygen, carbon dioxide related parameters in central and mixed venous samples and the effect of mechanical power on their values. Surprisingly, as shown in Figures 1, S1 and S2, PCO_2_, pH and bicarbonate concentration behaved similarly between central and mixed venous blood. On the opposite, central venous oxygen variables were only moderately correlated with their mixed venous equivalent (Figure 1). We may wonder how it is possible that oxygen incomplete mixing in right atrium (versus complete mixing in pulmonary artery) does not result in significant differences in the CO_2_‐related variables. One of the possible explanations is the different physical and biochemical behavior of O_2_ compared to the CO_2_. Indeed, oxygen binding to hemoglobin occurs almost completely inside red blood cells (RBCs), physical entities with their own volume. Indeed, ~ 98% of oxygen is linked to hemoglobin; therefore, a complete mixing requires time. In contrast, CO_2_ equilibrium may partially occur immediately in plasma, with an equilibration time in the order of milliseconds, comparable to the time required for CO_2_ to be exchanged in the venous capillaries. The highest percentage of CO_2_ is carried in the whole blood as bicarbonates, both in plasma (~ 95%) and inside the RBCs (~ 75%–85%) (Andrew, 2000a). An additional element that might merit consideration is the high carbon dioxide solubility, greater than the one of oxygen. A molecule of CO_2_ penetrates an aqueous membrane about 20 times as rapidly as a molecule of oxygen (Andrew, 2000b). Strictly considering dissolved CO_2_, it is considering that free PCO_2_ in plasma strictly depends on the strong ion difference (SID), that was not different between the two sites in our study. In extreme cases, with SID different on the two samples, this factor may play another important role, that we are not able to assess from our data.

Irrespective of the reasons of the differences between oxygenation and CO_2_, the possible clinical consequences are straightforward. Clearly, in the case of oxygen variables, the use of central venous sample compared to the mixed one may carry out some misinterpretation. Indeed, ScvO_2_ is largely used as a summary variable, which alerts the clinician if some of its determinants (i.e., cardiac output, VO_2_, Hb and arterial oxygen saturation) are out of normal physiological range. As arterial oxygen saturation is easily monitored by pulse‐oximetry, any change in venous SO_2_ below 65–75, with an arterial oxygen saturation from 95 to 100, indicates increased metabolism (VO_2_), hemodynamic impairment (decreased CO) or anemia. Obviously, whether S$\bar{v}$O_2_ is 65% and ScvO_2_ is 10% higher, clinical judgment may be impaired. Indeed, while 75% may be considered as a normal value not requiring any particular action, 65% is a value which should lead to a search of possible causes of altered VO_2_/DO_2_ relationship (Rivers et al., 2001). Accordingly, all oxygen‐derived variables, as VO_2_ or venous admixture, suffer the same limitations when computed using central venous blood samples.

In contrast, CO_2_‐related analytes measured with a central venous catheter may be used with a higher level of confidence as surrogates of mixed venous blood. Consequently, variables involving CO_2_ might be safely computed using central venous samples.

A significant source of error though comes from the therapeutic strategies delivered to patients – particularly mechanical ventilation. Specifically, the effect of positive pressure ventilation on venous return and hemodynamics in a way which is less predictable and may affect not just the absolute value of blood oximetry but also their trends overtime if mechanical ventilation settings vary in their intensity.

## 5 LIMITATIONS

The main limitations of our study are the retrospective and the pre‐clinical experimental design. Our results show that S_c_vO_2_ is always higher than S$\bar{v}$O_2_. This should limit the value of our observations to all situations in which the central oxygen saturation is higher than the mixed one.

The oxygen extraction ratio in our piglets is below the critical O_2_ER reported in the literature, with low values of venous admixture. Indeed, the study population includes healthy piglets, mechanically ventilated and with good tissue perfusion, leading to high oxygen delivery (DO_2_). This could somehow limit the consistency of our finding in patients with higher O_2_ER and Qva/Qt.

The cutoff of MP used in our study was set on the median value of all the observations and allowed us to have two balanced groups of mechanically ventilated piglets (39 in each group). However, this cutoff is higher when compared to the MP conventionally used in critically ill patients (Serpa Neto et al., 2018). Of note, however, all the observations made on MP and PEEP were confirmed with the results of the mixed linear models, considering MP and PEEP as continuous variables.

In conclusion, this study showed that the use of central venous sample to measure oxygenation variables increases the error risks. In contrast, central venous samples may be safely used as surrogates of mixed venous blood when assessing CO_2_‐derived variables. Similar caution should be exerted to mixed variables as ΔPCO_2_/ΔContO_2_ obtained from a central venous catheter. Indeed, in this case, ΔPCO_2_ might be considered reliable, while ΔContO_2_ could be somehow lower (up to 10%) than the one obtained by mixed venous blood. Therefore, the ratio could be consistently lower when measured by central instead of mixed venous blood and this ratio will be affected by the ventilatory settings in a less predictable way.

## AUTHOR CONTRIBUTIONS

Conceptualization: AF, TP, LG; Methodology: AF, TP, LG, IF, RMV; Software: AF, TP; Validation: FR, LG, OM, KM, LC; Formal Analysis: AF, TP, LG; Investigation: AF, TP, MB, FC; Resources: AF, TP, IF, RMV, MB, FC; Data Curation: AF, TP, IF, RMV, MB, FC; Writing – Original Draft preparation: AF, LG; Writing – Review & Editing: LC, FC, MB; Visualization: RMV, IF; Supervision: LG, LC, KM, OM; Project Administration: LG, FR; Funding Acquisition: This research received no external funding.

## CONFLICT OF INTEREST STATEMENT

The Authors declare no conflicts of interest.

## CONSENT

Not applicable.

## Supporting information


Data S1.

## Data Availability

All data are available in appropriate datasets.
