# Supplementary material for: Impact of mechanical power and positive end expiratory pressure on central vs. mixed oxygen and carbon dioxide related variables in a population of female piglets
Source: Physiol Rep. 2024 Feb 16;12(4):e15954. doi: 10.14814/phy2.15954 (PMC10873162; doi:10.14814/phy2.15954)
Supplement: Supplementary file 1 — Data S1. [file PHY2-12-e15954-s001.docx]

**Impact of Mechanical Power and Positive End Expiratory Pressure on central *vs* mixed oxygen and carbon dioxide related variables in a population of female piglets**

**Supplementary material**

***Index***

1. ***Supplementary figures***
   1. *Figure S1*
   2. *Figure S2*
   3. *Figure S3*
   4. *Figure S4*
   5. *Figure S5*
2. ***Supplementary tables***
   1. *Table S1*
   2. *Table S2*
   3. *Table S3*
   4. *Table S4*
   5. *Table S5*
3. ***Formulas***
   1. *Oxygen content (CxO_2_)*
   2. *Oxygen delivery (DO_2_)*
   3. *Oxygen consumption (VO_2_)*
   4. *Oxygen extraction ratio (O_2_ER)*
   5. *Venous Admixture (Qva/Qt)*
   6. *PCO_2_ gradient*
   7. *Bicarbonate concentration*
   8. *CO_2_ content*
   9. *Mechanical Power*
4. ***References***

***Figures***

*1.1 Figure S1*

**
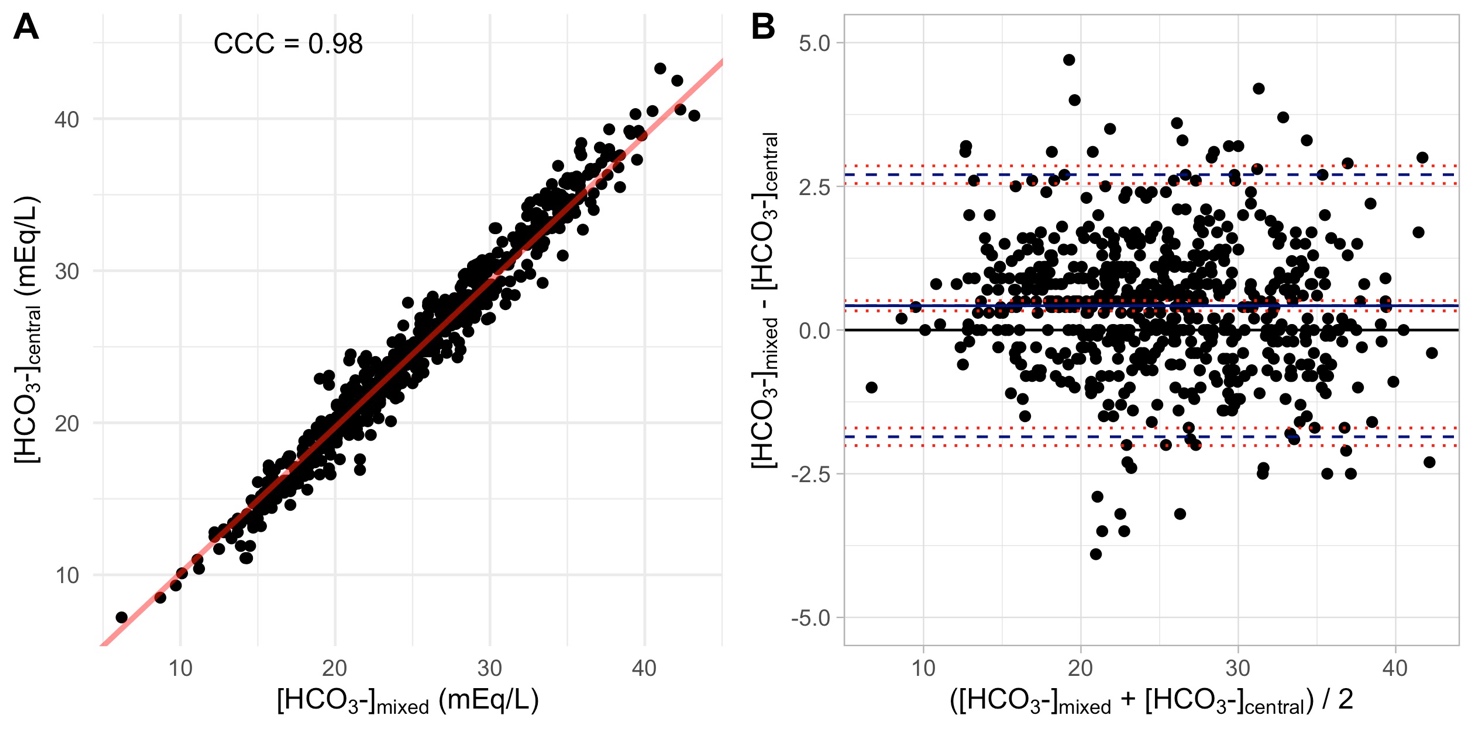
**

Figure S1. Panel A: Linear regression between bicarbonate concentration in mixed and central venous blood sample. [HCO_3_^-^]mixed and [HCO_3_^-^]central show a very strong correlation (CCC = 0.98). Panel B: Bland-Altman analysis between [HCO_3_^-^]mixed and [HCO_3_^-^]central, showing a mean bias of 0.42 [0.33 – 0.51], upper LOA = 2.70 [2.55 – 2.86], lower LOA = -1.85 [-2.01 - -1.70]. Legends: CCC = Concordance Correlation Coefficient; [HCO_3_^-^]mixed = mixed venous blood bicarbonates concentration; [HCO_3_^-^]central = central venous blood bicarbonates concentration; mEq/L = milliequivalents per Liter.

1.2 Figure S2

**
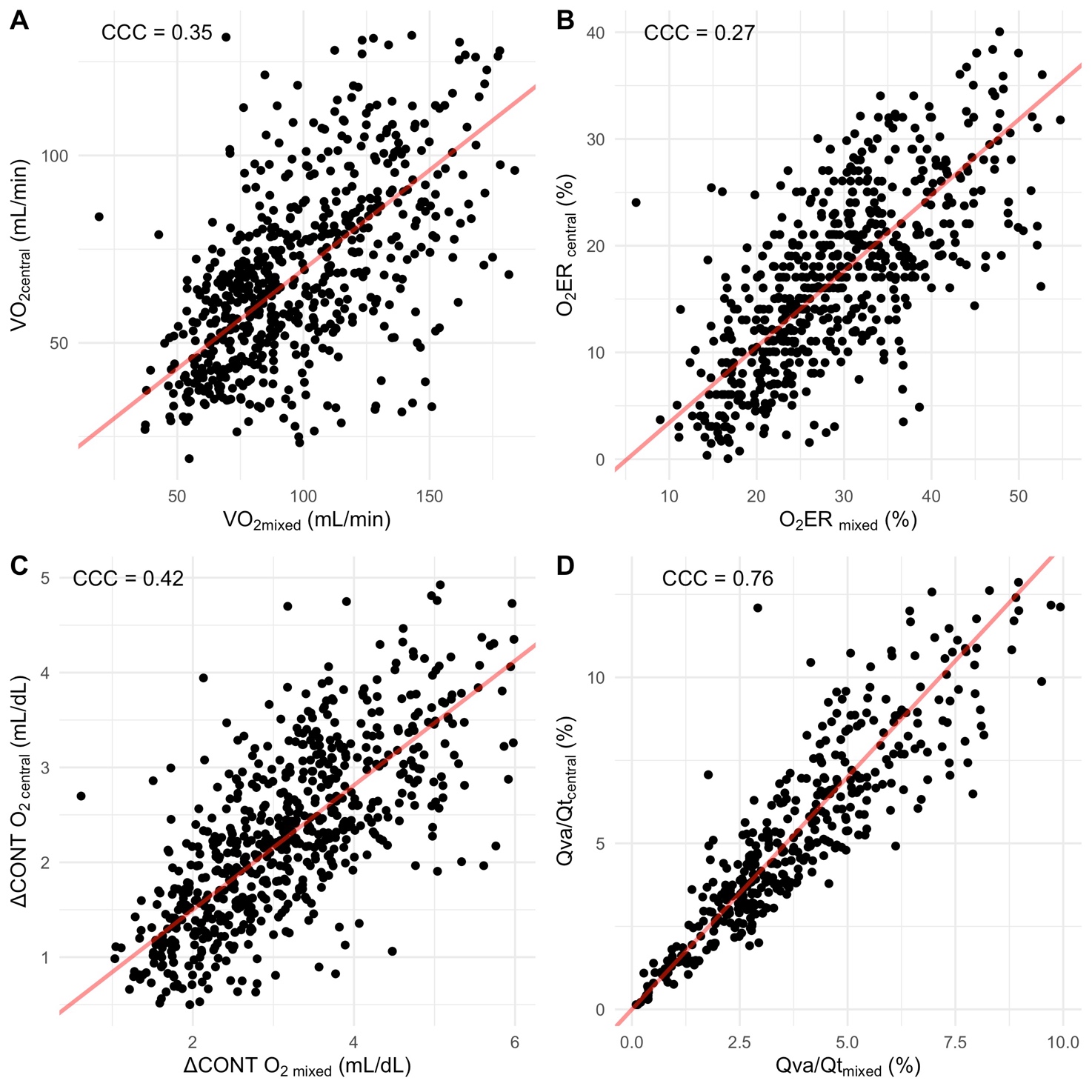
**

Figure S2. Panel A: Linear regression between VO_2_ in a mixed and a central venous blood sample. Panel B: Linear regression between O_2_ER in a mixed and a central venous blood sample. Panel C: Linear regression between ΔCont O_2_ in a mixed and a central venous blood sample. Panel D: Linear regression between Qva/Qt in a mixed and a central venous blood sample. Legends: CCC = Concordance Correlation Coefficient; VO_2mixed =_ Oxygen consumption from a mixed venous blood sample; VO_2central =_ Oxygen consumption from a central venous blood sample; O_2_ER_mixed_ = oxygen extraction ratio from a mixed venous blood sample; O_2_ER_central_ = oxygen extraction ratio from a central venous blood sample; ΔCont O_2 mixed_ = arteriovenous difference of oxygen content from a mixed venous blood sample; ΔCont O_2 central_ = arteriovenous difference of oxygen content from a central venous blood sample; [HCO_3_^-^]mixed = mixed venous blood bicarbonates concentration; Qva/Qt_mixed_= pulmonary venous admixture calculated with a mixed venous blood sample; Qva/Qt_central_= pulmonary venous admixture calculated with a central venous blood sample; % = percentage; mL/min = milliliters per minute; mL/dL = milliliters per deciliter of blood.

*1.3 Figure S3*

**
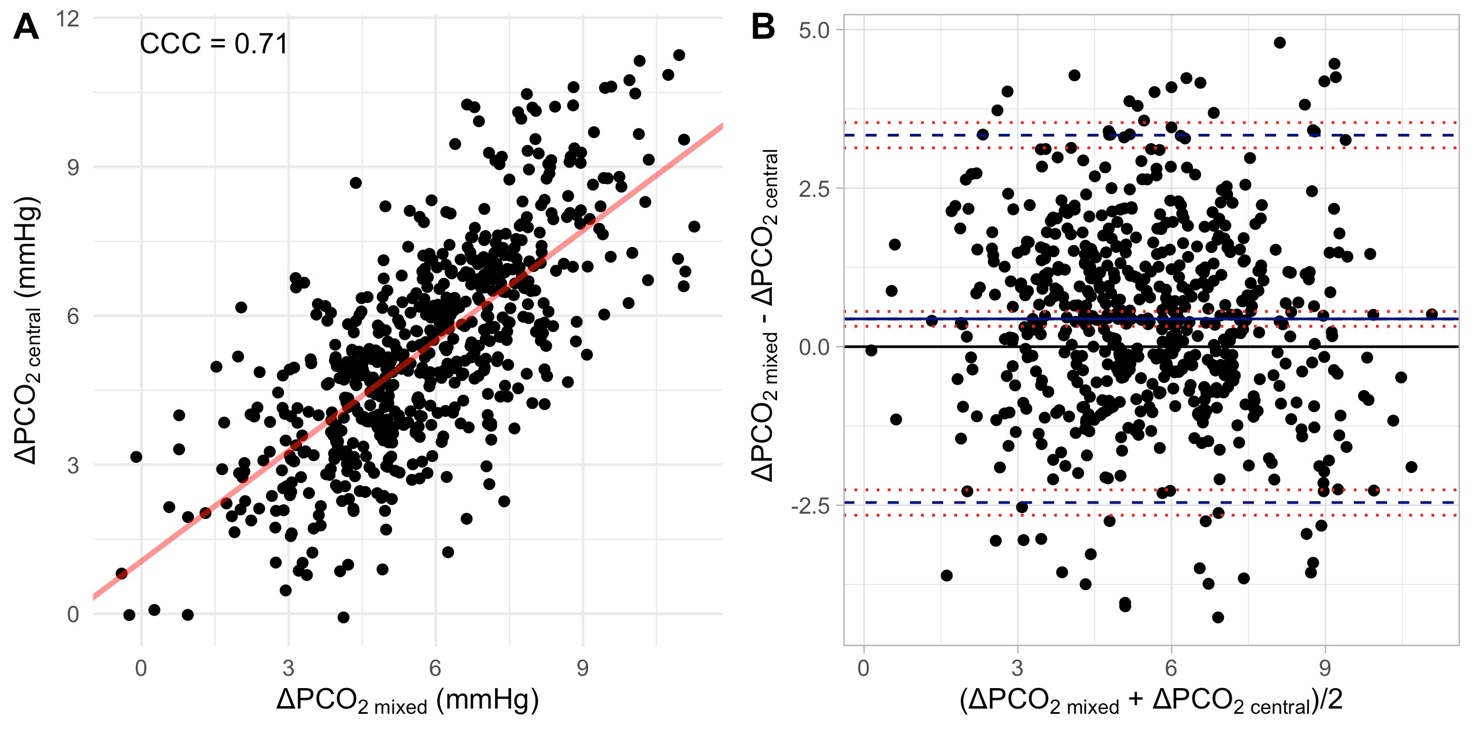
**

Figure S3. Panel A: Linear regression between ΔPCO_2_ in a mixed and a central venous blood sample. Panel B: Bland-Altman analysis between ΔPCO_2._ mixed and ΔPCO_2_ central, showing a mean bias of 0.44 [0.32 – 0.55], upper LOA = 3.33 [3.13 –3.53], lower LOA = -2.45 [-2.66 - -2.26]. Legends: CCC = Concordance Correlation Coefficient; ΔPCO_2._ mixed = veno-arterial carbon dioxide tension difference with a mixed venous blood sample; ΔPCO_2._ central = veno-arterial carbon dioxide tension difference with a central venous blood sample; mmHg = millimitres of mercury.

*1.4 Figure S4*

**
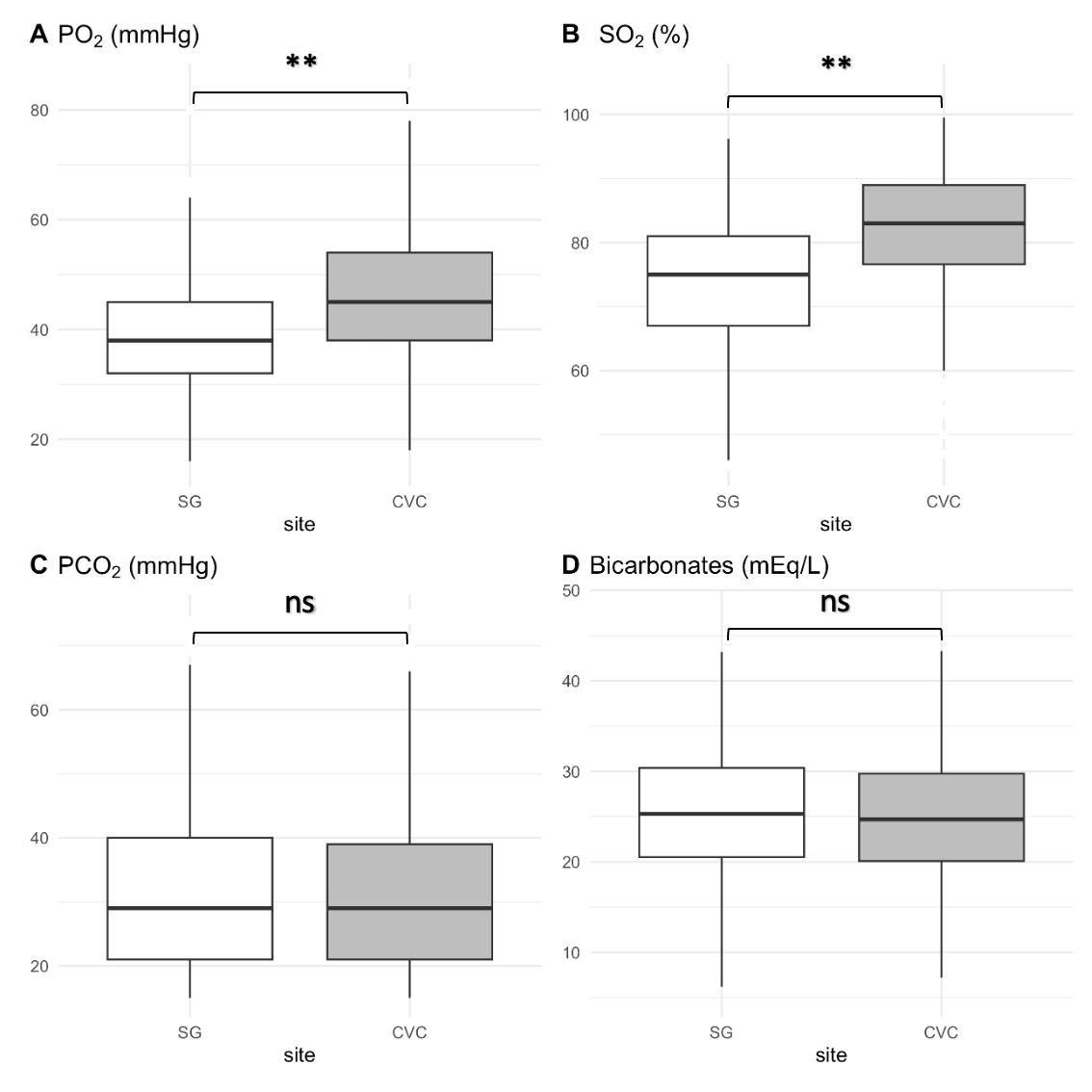
**

**p < 0.0001**

p = 0.191

p = 0.264

**p < 0.0001**

Figure S4. Boxplots showing median and interquartile range of PO_2_, SO_2_, PCO_2_ and bicarbonates in a mixed versus a central venous blood sample, from the whole population. White boxplots refer to value from the mixed venous sample, whilst gray samples refer to samples from a central venous blood sample. Legends: PO_2_ = Oxygen partial tension; SO2 = oxygen saturation; PCO2 = carbon dioxide partial tension; mmHg = millimitres of mercury; mEq/L = milliequivalents per liter.

*1.5 Figure S5*

**p = 0.023**

**p = 0.020**


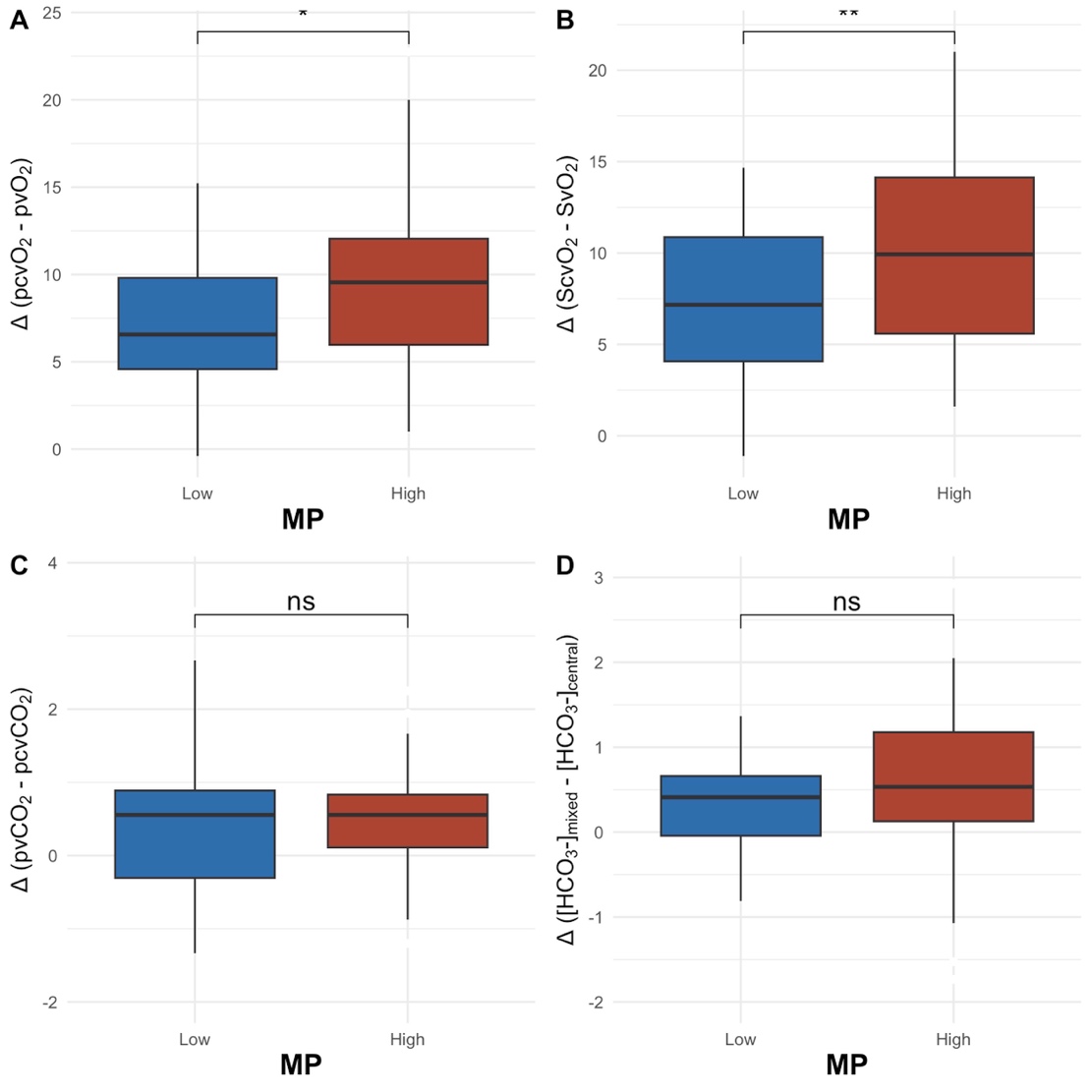


p = 0.113

p = 0.344

*Figure S5. Δ(P_c_vO_2_ - P*$\bar{v}$*O_2_), Δ(S_c_vO_2_ - S*$\bar{v}$*O_2_), Δ(p*$\bar{v}$*CO_2_ - PcvCO_2_), Δ([HCO_3_^-^]mixed - [HCO_3_^-^]central) in piglets ventilated with low versus high mechanical power. Blue and red boxplots refer respectively to piglets ventilated with low and high mechanical power. -. Legends: Δ(P_c_vO_2_ - P*$\bar{v}$*O_2_) = difference between central and mixed venous blood oxygen partial tension; Δ(S_c_vO_2_ - S*$\bar{v}$*O_2_)* = *difference between central and mixed venous blood oxygen saturation; Δ(p*$\bar{v}$*CO_2_ - PcvCO_2_) = difference between mixed and central carbon dioxide partial tension; Δ([HCO_3_^-^]mixed - [HCO_3_^-^]central) = difference between mixed and central venous blood bicarbonate concentration; MP = mechanical power. For numerical details, see also table S4 in the supplementary materials.*

*2.1 Table S1*

|  | Low MP  n = 39 | High MP  n = 39 | p |
| --- | --- | --- | --- |
| Pig weight (kg) | 23.8 $\pm$ 2.2 | 23.9 $\pm$ 2.1 | 0.860 |
| Length (cm) | 97.7 $\pm$ 4.5 | 97.3 $\pm$ 4.5 | 0.751 |
| Chest circumference (cm) | 61.5 $\pm$ 2.4 | 61.2 $\pm$ 2.7 | 0.704 |
| Temperature (°C) | 35.8 $\pm$ 1.2 | 35.9 $\pm$ 1.1 | 0.902 |
| Tidal Volume (mL) | 233 $\pm$ 30.1 | 238 $\pm$ 24.6 | 0.401 |
| PEEP (cmH_2_O) | 5 [5 – 5] | 5 [5 - 5] | 0.165 |
| Respiratory Rate (bpm) | 20 [20 – 22] | 20 [20 - 22] | 0.958 |
| Inspired fraction of oxygen | 0.4 [0.4 – 0.4] | 0.4 [0.4 – 0.4] | 0.330 |
| Mean Airway pressure (cmH_2_O) | 9 [8.8 - 10] | 9 [9 - 10] | 0.215 |
| Driving pressure (cmH_2_O) | 9.3 [8.1 – 10.3] | 8.9 [8.1 – 9.9] | 0.331 |
| Heart rate (bpm) | 96.8 $\pm$ 16.9 | 98.2 $\pm$ 20 | 0.811 |
| Mean Arterial Pressure (mmHg) | 74 [69.5 - 78] | 75 [70 - 76] | 0.822 |
| Central Venous Pressure (mmHg) | 7 [5 - 9] | 6 [4 - 8] | 0.134 |
| Systemic Vascular Resistances (dynes/seconds/cm^-5^) | 1393 [1142 - 1598] | 1234 [1154 - 1432] | 0.999 |
| Mean Pulmonary Arterial Pressure (mmHg) | 19.9 $\pm$ 3.5 | 20.2 $\pm$ 3.8 | 0.834 |
| Pulmonary Capillary Wedge Pressure (mmHg) | 10 [7 - 11] | 9 [8 - 10] | 0.345 |
| Pulmonary Vascular Resistances (dynes/seconds/cm^-5^) | 201 $\pm$ 71 | 211 $\pm$ 71 | 0.650 |
| Stroke Volume (mL) | 41 $\pm$ 7 | 40 $\pm$ 8 | 0.981 |
| Cardiac Output (L/min) | 4.1 $\pm$ 0.8 | 4.1 $\pm$ 0.7 | 0.917 |
| PaO_2_ (mmHg) | 219 $\pm$ 12.7 | 221 $\pm$ 12.5 | 0.491 |
| P$\bar{v}$O_2_ (mmHg) | 51.8 $\pm$ 4.9 | 50.3 $\pm$ 6.4 | 0.395 |
| PcvO_2_ (mmHg) | 64 $\pm$ 12.4 | 65.2 $\pm11.3$ | 0.725 |
| S$\bar{v}$O_2_ (%) | 84.0 [80.8 – 87.0] | 83.1 [79.6 – 85.9] | 0.583 |
| ScvO_2_ (%) | 92.0 [87.6 – 94.2] | 92.0 [86.8 – 94.4] | 0.774 |
| Δ(ScvO_2_ – S$\bar{v}$O_2_) (%) | 8.9 [5.3 – 12.4] | 9.3 [4 – 12.4] | 0.882 |
| P$\bar{v}$CO_2_ (mmHg) | 50.5 $\pm$ 4 | 50.6 $\pm$ 6 | 0.960 |
| PcvCO_2_ (mmHg) | 49 $\pm$ 4 | 49 $\pm$ 5 | 0.947 |
| Systemic Oxygen Delivery (DO_2_, mL/min) | 448 $\pm$ 111 | 466 $\pm$ 113 | 0.598 |
| Systemic Oxygen Consumption (VO_2_, mL/min) | 88 $\pm$ 22 | 466 ± 30 | 0.459 |
| Hemoglobin (g/dL) | 7.5 [6.9 – 8.2] | 7.8 [7.0 – 8.7] | 0.715 |
| Arterial pH | 7.51 [7.47 – 7.56] | 7.52 [7.49 – 7.54] | 0.782 |
| Blood lactates (mMol/L) | 0.5 [0.4 – 0.7] | 0.5 [0.4 – 0.9] | 0.445 |

*Table S1. Zoometric, respiratory, hemodynamic and metabolic variables at baseline. Variables with a normal distribution are displayes with their mean* $\pm$ *standard deviation. Variables non-normally distributed are displayed with their median and interquartile range (in square brackets). All the shown p-values are both-sided. Legends: °C: Celsius degree; cmH_2_O: centimeters of water; bpm: beats per minute; mmHg: millimeters of mercury; L: liters; min: minutes; S*$\bar{v}$*O_2_: mixed venous oxygen saturation; S_c_vO_2_: central venous oxygen saturation; O_2_ER_mixed_: oxygen extraction ratio calculated with the mixed venous sample; O_2_ER_central_: oxygen extraction ratio measured with the central venous sample. paO_2_: arterial oxygen partial pressure; mmol: millimols; g: grams.*

*2.2 Table S2*

| Variable | Time | | MP | | Time * MP | |
| --- | --- | --- | --- | --- | --- | --- |
|  | **β** | **p** | **β** | **p** | **β** | **p** |
| Δ (P_c_vO_2_ – PvO_2_) | -0.01 | 0.82 | 0.17 | **0.004** | -0.01 | 0.06 |
| Δ (S_c_vO_2_ – SvO_2_) | -0.05 | 0.25 | 0.13 | **0.05** | 0.0009 | 0.56 |
| Δ (PvCO_2_ – PcvCO_2_) | -0.04 | **0.006** | 0.0004 | 0.97 | 0.0004 | 0.36 |
| Δ ([HCO_3_^-^] mixed – [HCO_3_^-^] central]) | -0.009 | 0.26 | 0.02 | 0.135 | 0.0001 | 0.88 |

*Table S2: Linear mixed model showing the effect of time, mechanical power and their interaction (Time * MP) on the difference between mixed and central venous sample of oxygen and carbon-dioxide related variables. As shown, the mechanical power has a significant effect on oxygen related variables. Conversely, carbon dioxide related variables did not change accordingly to the mechanical power. All the shown p-values are both-sided. Legends: MP = mechanical power; β = beta; Δ(P_c_vO_2_ - P*$\bar{v}$*O_2_) = difference between central and mixed venous blood oxygen partial tension; Δ(S_c_vO_2_ - S*$\bar{v}$*O_2_)* = *difference between central and mixed venous blood oxygen saturation; Δ(p*$\bar{v}$*CO_2_ - PcvCO_2_) = difference between mixed and central carbon dioxide partial tension; Δ([HCO_3_^-^]mixed - [HCO_3_^-^]central) = difference between mixed and central venous blood bicarbonate concentration.*

*2.3 Table S3*

| Groups | Low/High | | p |
| --- | --- | --- | --- |
| **Mechanical Power** | Low (< 23.9 J/min) | High (> 23.9 J/min) |  |
| Δ (S_c_vO_2_ – SvO_2_) | 7.22 [3.9 – 10.9] | 10 [5.46-14.2] | **0.020** |
| **Tidal Volume** | Low (< 14.3 mL/kg) | High (> 14.3 mL/kg) |  |
| Δ (S_c_vO_2_ – SvO_2_) | 7.9 [4.4 – 11.8] | 9.1 [4.9 - 13.4] | 0.411 |
| **RR** | Low (< 30/min) | High (> 30/min) |  |
| Δ (S_c_vO_2_ – SvO_2_) | 9 [5.1 - 13] | 5.4 [1.4 -11.4] | 0.085 |
| **PEEP** | Low (< 5 cmH_2_O) | High (> 5 cmH_2_O) |  |
| Δ (S_c_vO_2_ – SvO_2_) | 6.2 [3.7 – 10.8] | 10.6 [7.1 - 14.2] | **0.001** |

*Table S3: Difference between the central and mixed venous sample (Δ(S_c_vO2 – S*$\bar{v}$*O2)) in pigs separated on the median value of Mechanical Power, Tidal Volume, Positive End Expiratory Pressure, Respiratory Rate, airways driving pressure, airways peak pressure. For each piglet, the mean value of all the Δ(S_c_vO2 – S*$\bar{v}$*O_2_) available during the experimental phase (from timepoint 0.5 hour to 48 hours) was considered. The Δ(S_c_vO2 – S*$\bar{v}$*O_2_) in the two groups is expressed as median and interquartile range (in square brackets). Comparisons were made via a Wilcoxon test. All p-values shown in the table are two-sided. Legends: J/min = Joules per minute; mL/kg = milliliters per kilogram; min = minute; cmH_2_O = centimeters of water; Δ(S_c_vO_2_ - S*$\bar{v}$*O_2_) = difference between central and mixed venous blood oxygen saturation; RR = Respiratory Rate; PEEP = Positive End Expiratory Pressure.*

*2.4 Table S4*

| Groups | Low/High Mechanical Power | | p |
| --- | --- | --- | --- |
|  | **Low (< 23.9 J/min)** | **High (> 23.9 J/min)** |  |
| **Δ (P_c_vO_2_ – PvO_2_)** | 6.56 [4.58 – 9.81] | 9.55 [5.97 – 12.00] | **0.023** |
| **Δ (S_c_vO_2_ – SvO_2_)** | 7.22 [3.9 – 10.9] | 10.00 [5.46-14.20] | **0.020** |
| **Δ (PvCO_2_ – PcvCO_2_)** | 0.56 [-0.30 – 0.89] | 0.56 [0.11 – 0.87] | 0.344 |
| **Δ ([HCO_3_^-^] mixed – [HCO_3_^-^] central])** | 0.41 [-0.04 – 0.66] | 0.55 [0.13 – 1.23] | 0.113 |

*Table S4: Effect of the Mechanical Power on oxygen and carbon-dioxide related variables. Two groups of Mechanical Power have been created, dividing the population on the median value of MP during the experimental phase (23.9 J/min). Comparisons were made via a Wilcoxon test. All the shown p-values are both-sided. Values are expressed as median and interquartile range (square brackets). Legends: J/min = Joules per minute; Δ(P_c_vO_2_ - P*$\bar{v}$*O_2_) = difference between central and mixed venous blood oxygen partial tension; Δ(S_c_vO_2_ - S*$\bar{v}$*O_2_)* = *difference between central and mixed venous blood oxygen saturation; Δ(p*$\bar{v}$*CO_2_ - PcvCO_2_) = difference between mixed and central carbon dioxide partial tension; Δ([HCO_3_^-^]mixed - [HCO_3_^-^]central) = difference between mixed and central venous blood bicarbonate concentration.*

*2.5 Table S5*

| MP groups | MP lowest quartile (< 13.7 J/min) | MP highest quartile (> 30.7 J/min) |
| --- | --- | --- |
| Δ (ScvO_2_ – S$\bar{\boldsymbol{v}}$O_2_) (%) | 8.7 $\pm$ 6.2 | 11.3 $\pm$ 8.1 |
| PEEP groups | PEEP lowest quartile (< 5 cmH_2_O) | PEEP highest quartile (> 14 cmH_2_O) |
| Δ (ScvO_2_ – S$\bar{\boldsymbol{v}}$O_2_) (%) | 6.8 $\pm$ 5.1 | 10.8 $\pm$ 7.7 |

*Table S5: Δ(S_c_vO_2_ - S*$\bar{v}$*O_2_)* *values in animals ventilated at the lowest/highest mechanical power and lowest/highest PEEP. Animals in the first and fourth quartile of mechanical power and PEEP were included. Mean and standard deviation of central versus mixed venous oxygen saturation in the four groups are shown*. *Legends: MP = Mechanical Power; J/min = Joules per minute; Δ(S_c_vO_2_ - S*$\bar{v}$*O_2_)* = *difference between central and mixed venous blood oxygen saturation; PEEP = Positive End-Expiratory Pressure; cmH_2_O = centimeters of water.*

***Formulas***

*3.1 Oxygen content (CxO_2_)*

$$CxO_{2}=1.36\times\left[ Hb \right]\times SxO_{2}+0.0031\times PxO_{2}$$

Where [Hb] is hemoglobin concentration in g/dL, SxO_2_ is oxygen saturation in % and PxO_2_ is oxygen partial pressure in mmHg. The subscript x denotes the sample site and can be arterial (CaO_2_, SaO_2_, PaO_2_), central venous (CcvO_2_, ScvO_2_, PcvO_2_) or mixed venous (CvO_2_, SvO_2_, PvO_2_).

Capillary oxygen content (CcO_2_) was calculated assuming a oxygen saturation of 100% and using alveolar PO_2_ (PAO_2_):

$$P_{A}O_{2}=713\times FiO_{2}-\frac{PaCO_{2}}{R}$$

Where FiO_2_ is the inspired oxygen fraction, PaCO_2_ is arterial carbon dioxide partial pressure and R is respiratory quotient, assumed to be 0.8.

*3.2 Oxygen delivery (DO_2_)*

$$DO_{2}=CaO_{2}\times CO\times10$$

Where CaO_2_ is the arterial oxygen content in mL/dL and CO is cardiac output in L/min.

*Oxygen consumption (VO_2_)*

Fick equation was used to calculate VO_2_:

$$VO_{2}=(CaO_{2}-CxO_{2})\times CO\times10$$

Where CaO_2_ is the arterial oxygen content in mL/dL, CxO_2_ can be central venous (CcvO_2_) or mixed venous oxygen content (CvO_2_) in mL/dL, CO is cardiac output in L/min. Accordingly,

$$VO_{2 mixed}=(CaO_{2}-CvO_{2})\times CO\times10$$

$$VO_{2 central}=(CaO_{2}-CcvO_{2})\times CO\times10$$

*3.3 Oxygen extraction ratio (O_2_ER)*

$$O_{2}ER= \frac{{VO}_{2}}{{DO}_{2}}=\frac{({CaO}_{2}-{CxO}_{2}) \times CO\times10}{{CaO}_{2}\times CO\times10}$$

Where VO_2_ is oxygen consumption, DO_2_ is oxygen delivery, CaO_2_ is the arterial oxygen content in mL/dL, CxO_2_ can be central venous (CcvO_2_) or mixed venous oxygen content (C$\bar{v}$O_2_) in mL/dL, CO is cardiac output in L/min. Accordingly,

$$O_{2}ER_{mixed}= \frac{VO_{2 mixed}}{{DO}_{2}}=\frac{({CaO}_{2}-{C\bar{v}O}_{2}) \times CO\times10}{{CaO}_{2}\times CO\times10}$$

$$O_{2}ER_{central}= \frac{VO_{2 central}}{{DO}_{2}}=\frac{({CaO}_{2}-{CcvO}_{2}) \times CO\times10}{{CaO}_{2}\times CO\times10}$$

*3.4 Venous admixture (Qva/Qt)*

Berggren equation was used to calculate venous admixture:

$$\frac{Qva}{Qt}=\frac{CcO_{2}-CaO_{2}}{CcO_{2}-CxO_{2}}\times100$$

Where CcO_2_ is capillary oxygen content in mL/dL, CaO_2_ is arterial oxygen content in mL/dL and CxO_2_ can be central venous (CcvO_2_) or mixed venous oxygen content (C$\bar{v}$O_2_) in mL/dL. Accordingly,

$${Qva}/{Qt_{mixed}}=\frac{CcO_{2}-CaO_{2}}{CcO_{2}-C\bar{v}O_{2}}\times100$$

$${Qva}/{Qt_{ventral}}=\frac{CcO_{2}-CaO_{2}}{CcO_{2}-CcvO_{2}}\times100$$

*3.5 PCO_2_ gradient*

$$\Delta PxaCO_{2}=PxCO_{2}-PaCO_{2}$$

Where PxCO_2_ can be central venous (PcvCO_2_) or mixed venous carbon dioxide partial pressure (PvCO_2_) in mmHg. Accordingly,

$$\Delta PCO_{2 mixed}=P\bar{v}CO_{2}-PaCO_{2}$$

$$\Delta PCO_{2 central}=PcvCO_{2}-PaCO_{2}$$

*3.6 Bicarbonate concentration*

$$\left[ HCO_{3}^{-} \right]=0.0306\times PxCO_{2}\times{10}^{(pH-6.1)}$$

Where PxCO_2_ can be arterial (PaCO_2_), central venous (PcvCO_2_) or mixed venous carbon dioxide partial pressure (PvCO_2_) in mmHg; pH is relative to PCO_2_ sample site. Accordingly,

$$\left[ HCO_{3}^{-} \right]_{arterial}=0.0306\times PaCO_{2}\times{10}^{(pH-6.1)}$$

$$\left[ HCO_{3}^{-} \right]_{mixed}=0.0306\times P\bar{v}CO_{2}\times{10}^{(pH-6.1)}$$

$$\left[ HCO_{3}^{-} \right]_{central}=0.0306\times PcvCO_{2}\times{10}^{(pH-6.1)}$$

*3.7 CO_2_ content*

Arterial (CaCO_2_), central venous (CcvCO_2_) and mixed venous (C$\bar{v}$CO_2_) carbon dioxide content were calculated according to Douglas equation ^1^, using pH, PCO_2_, [Hb] and SO_2_ from respective samples.

*3.8 Mechanical Power (MP)* ^2^

$$MP =0.098 x RR x Vt x \left( Paw peak-\frac{Paw plat-PEEP}{2} \right)$$

Where:
0.098 = conversion factor from *cmH_2_O * l /min* to *J/min*

RR = respiratory rate (*x/min*)

Vt = Tidal Volume (*L*)

Paw Peak = Respiratory system peak pressures (*cmH_2_O*)

Paw Plat = Respiratory system plateau pressures (*cmH_2_O*)

PEEP = Positive End Expiratory Pressure (*cmH_2_O*)

**REFERENCES**

1. Douglas AR, Jones NL, Reed JW. Calculation of whole blood CO2 content. *J Appl Physiol (1985)*. 1988;65(1):473-477. doi:10.1152/JAPPL.1988.65.1.473

2. Chiumello D, Gotti M, Guanziroli M, et al. Bedside calculation of mechanical power during volume- And pressure-controlled mechanical ventilation. *Crit Care*. 2020;24(1):1-8. doi:10.1186/S13054-020-03116-W/FIGURES/3
